# Apoplastic and symplastic phloem loading in *Quercus robur* and *Fraxinus excelsior*

**DOI:** 10.1093/jxb/eru066

**Published:** 2014-03-03

**Authors:** Soner Öner-Sieben, Gertrud Lohaus

**Affiliations:** Molekulare Pflanzenforschung/Pflanzenbiochemie, Bergische Universität Wuppertal, Gaußstraße 20, D-42119 Wuppertal, Germany

**Keywords:** *Fraxinus excelsior*, non-aqueous fractionation, phloem loading, *Quercus robur*, subcellular metabolite concentration, sucrose transporter, sugar.

## Abstract

Whereas most of the research on phloem loading is performed on herbaceous plants, less is known about phloem loading strategies in trees. In this study, the phloem loading mechanisms of *Quercus robur* and *Fraxinus excelsior* were analysed. The following features were examined: the minor vein structure, the sugar concentrations in phloem sap by the laser–aphid–stylet technique, the distribution of photoassimilates in the mesophyll cells by non-aqueous fractionation, gradients of sugar concentrations and osmotic pressure, and the expression of sucrose transporters. The minor vein configurations of *Q. robur* and *F. excelsior* belong to the open type. *Quercus robur* contained companion cells in the minor veins whereas *F. excelsior* showed intermediary cells in addition to ordinary companion cells. The main carbon transport form in *Q. robur* was sucrose (~1M). In *F. excelsior* high amounts of raffinose and stachyose were also transported. However, in both tree species, the osmolality of phloem sap was higher than the osmolality of the mesophyll cells. The concentration gradients between phloem sap and the cytoplasm of mesophyll cells for sucrose were 16-fold and 14-fold for *Q. robur* and *F. excelsior*, respectively. Independent of the type of translocated sugars, sucrose transporter cDNAs were cloned from both species. The results indicate that phloem loading of sucrose and other metabolites must involve active loading steps in both tree species. *Quercus robur* seems to be an apoplastic phloem loader while *F. excelsior* shows indications of being a symplastic or mixed symplastic–apoplastic phloem loader.

## Summary

Concentration gradients of sugars between the cytosol of mesophyll cells and the phloem reveal that *Quercus robur* is probably an apoplastic phloem loader and *Fraxinus excelsior* a mixed phloem loader.

## Introduction

The sharing of work between autotrophic source tissues and heterotrophic sink organs in higher plants makes an efficient vascular system necessary to transport carbohydrates and other metabolites. In higher plants, long-distance transport of assimilates takes place in the phloem, and phloem transport is dependent on the loading of the phloem with sugars in source tissues and the removal of sugars from the phloem into the sink tissues. Several factors determine the mode of phloem loading in leaves: (i) the organization of the interface between the mesophyll and phloem (Gamalei, 1989) and the type of companion cells (CCs; minor vein structure); (ii) the types of transport sugars (Zimmermann and Ziegler, 1975); (iii) the existence of sucrose uptake transporters in the phloem (Riesmeier *et al.*, 1992; Sauer and Stolz, 1994); (iv) the sensitivity of phloem loading to *p*-chloromercuribenzensulphonic acid (PCMBS) (van Bel *et al.*, 1994); and (v) the existence of concentration gradients between the mesophyll cells (MCs) and the phloem (Lohaus *et al.*, 1995; Nadwodnik and Lohaus, 2008).

Most studies about phloem loading have been performed on herbaceous plants which load the phloem mainly actively, either apoplastically or symplastically, or a mixture of both (Giaquinta, 1976; Turgeon and Gowan, 1990; Riesmeier *et al.*, 1992; van Bel *et al.*, 1992; Sauer and Stolz, 1994; Voitsekhovskaja *et al.*, 2009). The potential of the plant to use a symplastic and/or apoplastic route for phloem loading is indicated by the abundance of plasmodesmal connections between phloem CCs and bundle sheath cells (BSCs). Based on this feature, Gamalei (1989) grouped plants into two main types: type 1 (open type) has the most numerous plasmodesmata in the minor vein phloem, and type 2 (closed type) has the fewest. Two types of CCs within these groups show the highest degree of specialization. These are intermediary cells (ICs; Turgeon *et al.*, 1993) with highly developed plasmodesmal connections to BSCs (belonging to open type 1) and transfer cells (TCs; Pate and Gunning, 1969) that have very few plasmodesmata (belonging to closed type 2). In type 2 species, phloem loading is apoplastic because of the symplastic isolation and depends on the activity of sugar transport proteins in the membranes, whereas in type 1 species phloem loading is symplastic through the plasmodesmata. Apoplastic phloem loaders transport nearly exclusively sucrose in the phloem, or sucrose together with sugar alcohols (Lohaus *et al.*, 1995; Nadwodnik and Lohaus, 2008). Sucrose uptake transporters as well as sugar alcohol transporters have been cloned (Riesmeier *et al.*, 1992; Sauer and Stolz, 1994; Ramsperger-Gleixner *et al.*, 2004) and shown to be expressed in the minor veins, which is in accordance with their supposed role in transporting their respective substrate from the apoplast into the phloem (Stadler *et al.*, 1995; Stadler and Sauer, 1996; Kühn *et al.*, 1997). Symplastic phloem loaders translocate high amounts of raffinose and stachyose in addition to sucrose (Voitsekhoskaja *et al*., 2006). The existence of these oligosaccharides was explained by the polymer trap model (Turgeon and Gowan, 1990). According to this model, raffinose and stachyose are synthesized from sucrose and galactinol in ICs. Furthermore, it was postulated that the size exclusion limit of plasmodesmata connecting ICs to the BSCs enables the passage of disaccharides such as sucrose from the mesophyll into the phloem, whereas the tri- and tetrasaccharides raffinose and stachyose remain trapped in the phloem. Both types of phloem loading, apoplastic and symplastic, result in much higher sugar concentrations in the phloem sap compared with the MCs and therefore phloem loading takes place against a concentration gradient (Lohaus *et al.*, 1995; Voitsekhoskaja *et al*., 2006). Several results indicated that at least some plants harbour redundant phloem loading strategies by the combination of active symplastic and apoplastic and/or passive loading mechanisms (Fisher, 1986; van Bel *et al.*, 1992; Voitsekhovskaja *et al.*, 2006; Slewinski *et al.*, 2013).

Minor veins that belong to the open type 1 group are heterogeneous in regard to the CCs. Therefore, the type 1 group was further divided into type 1 (IC) with intermediary cells with abundant, highly branched plasmodesmata that are typical for raffinose oligosaccharide (RFO) loaders and type 1 (CC) with ordinary CCs, but with a higher frequency of symmetrical plasmodesmata on the MC or BSC interface compared with CCs from type 2 minor veins (Davidson *et al.*, 2011). Minor vein type 1 (CC) should correspond to passive phloem loading, which is the third postulated loading mechanism, and several tree species fall into this subcategory (Gamalei, 1991; Rennie and Turgeon, 2009; Davidson *et al.*, 2011). Passive phloem loading is characterized as ‘thermodynamically downhill at the companion cell interface; transport compounds follow their concentration gradient into the companion cell’ (Slewinsky *et al.*, 2013). This loading mode can only occur if the solute concentration in the phloem is lower than the concentration in the MCs (Rennie and Turgeon, 2009; Slewinski *et al.*, 2013). Supporting the passive loading hypothesis, autoradiographs showed no accumulation of ^14^C-labelled sugars in minor veins of some tree leaves, whereas autoradiographs of herbs showed accumulation in veins compared with the surrounding tissue (Eschrich and Fromm, 1994; Turgeon and Medville, 1998). However, accumulation of sugars could be detected in ^14^C-images of tulip tree (*Lirodendron tulipifera*) leaves after the removal of the epidermis (Goggin *et al.*, 2001). The uptake of ^14^C is an approved method to distinguish between putative active and passive phloem loaders. However, because some woody plants tend to have much thicker leaves than herbs, this method leads to variable results in some cases. Therefore, additional parameters have to be taken into account in regard to phloem loading strategies (Goggin *et al.*, 2001). In addition, active phloem loading has also been shown in other type 1 (CC) tree species such as *Clethra barbinervis* or *Liquidambar styraciflua*, and the pathway is probably apoplastic (Turgeon and Medville, 2004). These results contradict the assumption that loading strategies can be determined entirely by minor vein structures. Probably in the large and diverse group of tree species several loading strategies exist.

As described above, the pathway and mechanism (active or passive) of phloem loading in tree species that belong to the open type 1 category are still a matter of debate. Active phloem loading describes the transport of sugars from the MCs into the phloem against a concentration gradient, whereas passive loading can only occur when the solute concentration in the phloem is lower than the concentration in the MCs. In order to decide whether passive or active mechanisms are involved in phloem loading, it is important to determine the concentration of sugars in the cytosol of mesophyll cells and in the phloem. Much of the information concerning subcellular metabolite concentrations in mesophyll cells is derived from studies on herbaceous plant species. In this study, the subcellular concentrations of sugar alcohols and mono-, di-, and oligosaccharides in *Quercus robur* (Fagaceae) and *Fraxinus excelsior* (Oleaceae) as well as the corresponding concentrations in the phloem sap were examined. These tree species were selected because *Q. robur* is a representative of the minor vein type 1 (CC) (Gamalei, 1989) and translocates sucrose (Zimmermann and Ziegler, 1975), whereas *F. excelsior* represents the minor vein type 1 (IC) (Gamalei, 1989) and translocates sucrose, RFOs, and mannitol (Zimmermann and Ziegler, 1975). Both species commonly occur in temperate forests. Based on the subcellular distribution, the concentration gradients of the metabolites in the different compartments which are involved in phloem loading were calculated. The nature of such metabolite gradients can give important insights into the mechanisms by which phloem loading for different metabolites may occur. Together with results from morphological studies of the minor vein structure and the expression of sucrose transporters (SUTs), the results are compared with predictions based on models of passive and active symplastic or apoplastic phloem loading that are currently discussed.

## Materials and methods

### Plant materials

*Quercus robur* and *Fraxinus excelsior* were grown in 5 litre pots in compost soil in a greenhouse. Three-year-old plants were used for the experiments. Leaf samples were harvested in June and July after exposure to 8h of illumination.

### Non-aqueous fractionation of leaves

The chosen method is based on the comparison of metabolite and marker enzyme distributions in the vacuolar, choroplastic, and cytoplasmic compartments (Riens *et al.*, 1991). For this analysis, a leaf was treated as a uniform metabolic compartment representing an average MC, subdivided into the three compartments listed above. Leaf samples were ground with a mortar and pestle to a fine powder in liquid nitrogen and lyophilized (Christ alpha 2–4, Martin Christ, Osterode am Harz, Germany). The procedure was conducted according to Riens *et al.* (1991) and Nadwodnik and Lohaus (2008). For *Q. robur*, density gradients between 1.33g ml^–1^ and 1.50g ml^–1^ and for *F. excelsior* between 1.35g ml^–1^ and 1.53g ml^–1^ were used. Six fractions were collected, aliquots of which were taken for the determination of the marker enzymes NADP-glyceraldehyde phosphate dehydogenase, chlorophyll, phosphoenolpyruvate carboxylase, and α-mannosidase as well as nitrate as markers for the chloroplast, cytosol, and vacuole, respectively (Klie *et al.*, 2011), and of metabolites. The cytosolic compartment was found to be enriched in the middle region of the gradient, the chloroplast material appeared in the region of lowest density, whereas the vacuolar material was mainly found in the fraction of highest density. For determination of sugar concentrations in the gradient fractions, chloroform:methanol extracts were prepared (see below). For the evaluation of the subcellular distribution of sugars between the stromal, cytoplasmic, and vacuolar compartment, a calculation according to Riens *et al.* (1991) was carried out.

### Extraction of sugars

The dried fractions of the gradients were used for the extraction of sugars. A 5ml aliquot of chloroform:methanol (1.5:3.5, v/v) was added to the pellet, and the sample was homogenized and kept on ice for 30min. The homogenate was then extracted twice with 3ml of water. The aqueous phases were combined and evaporated in a rotatory evaporator (RV 10 Digital, IKA, Staufen, Germany). The dried residue was dissolved in 1ml of ultrapure H_2_O (Millipore, Billerica, MA, USA), syringe-filtrated (0.20 μm nylon; Carl Roth, Germany), and stored at –80 °C until analysis.

### Collection of sieve tube sap

Sieve tube sap was obtained from severed stylets of aphids. For this method, aphids found on other tree leaves were taken. Approximately 10 aphids were caged for ~5h on the leaf of the experimental plant. Their stylets were cut by a laser beam (Lohaus *et al.*, 1995). The samples were collected with a micro capillary and subsequently ejected into 50 μl of ultrapure H_2_O (Millipore) and stored at –80 °C.

### Extraction of apoplastic wash fluids from leaves

The apoplastic wash fluids were collected according to Nadwodnik and Lohaus (2008) and were stored at –80 °C until analysis.

### Metabolite analysis

Sugars and sugar alcohols in tissue extracts, apoplastic fluid, and phloem sap were analysed by HPLC according to Nadwodnik and Lohaus (2008).

### Determination of the osmolality of the leaf sap

Discs were cut from leaves, placed in 2ml Eppendorf tubes, and frozen at –20 °C, then thawed on ice and centrifuged to extract cell sap from the tissue. This sap was used for the determination of the osmolality using the osmometer Wescor 5100B (Logan, UT, USA).

### Electron microscopy

Transmission electron microscopy was performed in cooperation with the Core Facility institute of Dr Zanger at the University of Düsseldorf, Germany. The cross-sectional areas of the subcellular compartments were quantified with analysis software (IMAGE J; public domain software, developed at the US National Institutes of Health, available at http://rsbweb.nih.gov/ij/)

### Preparation of total RNA

RNA from whole leaves was isolated using a modified protocol from Chang *et al.* (1993). A 100–200mg aliquot of leaf material was used. Integrity was checked by agarose gel electrophoresis and concentration was measured at 260nm wavelength.

### Synthesis of cDNA and PCR

First-strand cDNA was synthesized from 1ng of total RNA isolated from mature leaves using the ReverdAid™ First Strand cDNA Synthesis Kit (Fermentas, St. Leon-Rot, Germany) with an oligo(dT)_18_ primer. The single-stranded cDNA was used for PCR with the following primers: forward, 5’-GCI GCI GGI RTI CAR TTY GGI TGG GC-3’; reverse, 5’-GCI ACR TCI ARD ATC CAR AAI CC-3’ (Knop *et al.*, 2001). The degenerate primers were designed from two conserved regions that are ~330bp apart from each other, using sequence data from the published amino acid sequences of several sucrose uptake transporters (Knop *et al.*, 2001). To validate the sequences of the fragments, a BLASTX search was performed (http://blast.ncbi.nlm.nih.gov/Blast.cgi). Applying these fragments to rapid amplification of cDNA ends (RACE; 5’/3’ RACE Kit, Roche, Mannheim, Germany), full-length clones of putative sucrose transporters, named *FeSUT1* (accession no. KF736981) and *QrSUT1* (accession no. KF736982), were obtained. The open reading frames (ORFs) of the full sequences were phylogenetically analysed and compared with those of known SUTs using the MEGA software (Version 5, Build 5130517; http://www.megasoftware.net/) (Tamura *et al.*, 2011).

## Results

### Minor vein structure

**Fig. 1.**
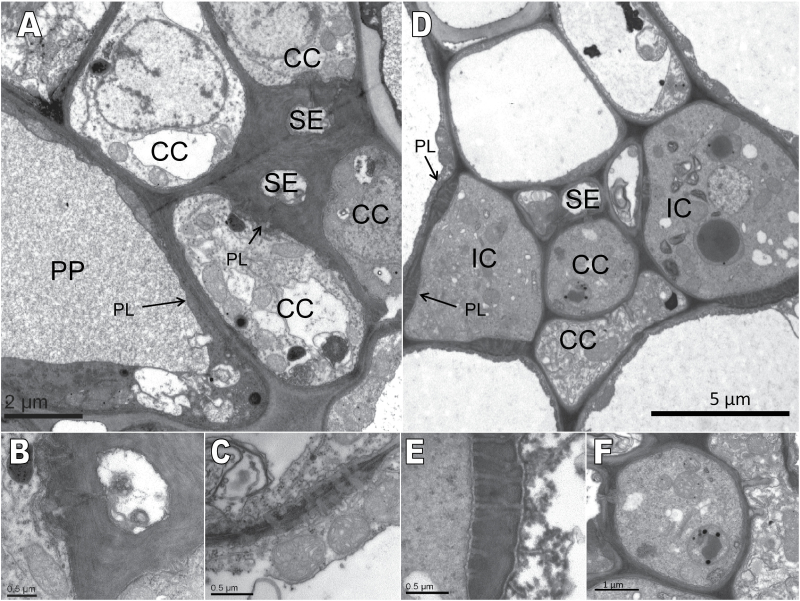
Electron micrographs of *Quercus robur* and *Fraxinus excelsior* minor veins and structures. CC, companion cell; SE, sieve element; IC, intermediary cell; PP, phloem parenchyma cell; PL and arrows indicate plasmodesmata. (A–C) Minor vein of *Q. robur*. (A) Minor vein configuration; (B) plasmodesmal connection between a CC and SE; (C) symmetrical PL between a CC and PP. The image was taken from another CC and PP pair because PL at this interface in image (A) were not distinct. (D–F) Minor vein of *F. excelsior*. (D) Minor vein configuration; (B) branched PL between an IC and adjacent cells such as a PP or bundle sheath cell; (F) an ordinary CC with plasmodesmal connection to an SE.

The minor vein structure was analysed to confirm the classifications into different minor vein types of *Q. robur* and *F. excelsior* performed by Gamalei (1989). The sieve elements (SEs) in the phloem of *Q. robur* were surrounded by CCs and phloem parenchyma cells (Fig. 1A). The SEs of minor veins were thick walled, whereas larger veins also contained thin-walled SEs (data not shown). CCs were joined to the SEs by branched plasmodesmata (Fig. 1B) which has already been shown for this interface in other species (Evert, 1990). Plasmodesmata between phloem parenchyma cells and CCs were symmetrically branched (Fig. 1C) and did not occur in dense fields. CCs were densely cytoplasmic, with small or sometimes larger vacuoles and many mitochondria.

In the minor vein of *F. excelsior*, two types of CCs were present: ICs and ordinary CCs (Fig. 1D). The minor vein was flanked by two ICs. They showed dense cytoplasm, many small vacuoles, and extremely abundant plasmodesmata that connected them to adjacent BSCs (Turgeon *et al.*, 1993). These plasmodesmata occurred in cluster and they had more branches on the IC side than on the BSC side (Fig. 1E). The ordinary CC (Fig. 1F) was connected to the SE by plasmodesmata, but no further plasmodesmatal connections to the adjacent cells were found. The cytoplasm of this type of CC was dense, the vacuoles were variable in size, and the nucleus occupied a relatively large portion of the cell volume. In the adaxial position, one or more parenchyma cells were commonly found, separating the SEs from the xylem vessels (Fig. 1D).

### Subcellular distributions of metabolites in leaves

For the analysis of possible phloem loading strategies it is important to determine the sugar concentrations in the cytosol of MCs and in the phloem. Cytosolic sugar contents can be determined by non-aqueous fractionation (Riens *et al.*, 1991). The further conversion of the cytosolic sugar contents into concentrations requires an estimation of the volume of the compartments (see below). Overall knowledge about the sugar distribution in MCs at the subcellular level (vacuolar, chloroplastic, and cytosolic compartment) allows the validity of the data to be checked comparing them with previous results. It should be mentioned that the non-aqueous fractionation technique does not resolve between the cytosol and endomembrane compartments. Therefore, the compartment was designated to the cytoplasm, which includes mitochondria, peroxisomes, and endoplasmic reticulum.

The subcellular distribution of hexoses and sucrose as measured by non-aqueous fractionation in *Q. robur* are shown in Table 1. Sugar alcohols such as mannitol or sorbitol and RFOs were not found in leaves of this species. The subcellular distribution of hexoses and sucrose was very different. Sucrose was located in the vacuole and the cytoplasm, whereas glucose and fructose were located mainly in the vacuole (Table 1).

**Table 1.** Content and percentage distribution of sugars among the vacuolar, stromal, and cytoplasmic compartments of leaves from Quercus robur and Fraxinus excelsiorData are mean values from four independent fractionations ±SD.

	Whole-leaf content (μmol g^–1^ FW)	Vacuole (%)	Stroma (%)	Cytoplasm (%)
*Quercus robur*				
Mannitol	ND			
Glucose and fructose	9.2±2.8	82.7±8.9	1.9±3.8	15.6±9.1
Sucrose	14.6±3.9	52.8±8.2	9.0±6.8	38.3±8.3
Raffinose	ND			
Stachyose	ND			
*Fraxinus excelsior*				
Mannitol	14.4±1.3	76.7±1.5	20.7±1.2	2.0±2.6
Glucose and fructose	9.1±3.0	85.0±17.3	3.5±6.0	11.5±12.0
Sucrose	5.9±1.3	73.0±22.9	0.3±0.6	26.7±22.3
Raffinose	0.5±0.2	80	20	0
Stachyose	1.3±0.5	80	20	0

ND, not detected.

*Fraxinus excelsior* contained mannitol and oligosaccharides in addition to hexoses and sucrose (Table 1). The subcellular distribution of mannitol was very different from the distribution of hexoses and sucrose. Mannitol was located mainly in the vacuole (77%) followed by the stroma of the chloroplasts (21%). In contrast, glucose and fructose were mainly located in the vacuole (85%) and sucrose was mainly distributed among the vacuolar (73%) and the cytoplasmic compartments (27%). The concentration of oligosaccharides in whole leaves was low [1.8 μmol g^–1^ fresh weight (FW)]. This was probably the reason why it was not possible to assign raffinose and stachyose to particular compartments in each experiment (Table 1). Therefore, only a rough allocation is shown in Table 1. The main portion of raffinose and stachyose was located in the vacuole and a lower portion in the chloroplast.

### Subcellular volumes

The further conversion of subcellular contents based on grams fresh weight (Table 1) into concentrations required knowledge of the relative volumes of the subcellular compartments. A determination was done by morphometric analysis of light and electron micrographs. Supplementary Table S1 available at *JXB* online shows the relative volumes of the vacuolar, chloroplastic, cytoplasmic (sum of cytosol, peroxisomes, mitochondria), and nuclear compartments of the MCs. In both species, the vacuole was the largest compartment within the cells and occupied 39.3–71.7% of the total volume, followed by the chloroplasts with 15.9–31.8%, the cytoplasm with 8.6–21.0%, and the nucleus with 3.9–7.8%. The relative portion of mitochondria and peroxisomes of the cytoplasm was ~13–15%, and the portion of the stroma of the chloroplast ranged from 48% to 58% (Winter *et al.*, 1993). In dicots, the main part of the aqueous volume of the leaf is occupied by the MCs (Winter *et al.*, 1993). The relative volume of the sieve tubes/CCs of the aqueous space of the leaf is <2% (Winter *et al.*, 1993). Hence, in *Q. robur* leaves with an average water content of 534 μl g^–1^ FW, the volumes of the vacuolar, stromal, and cytoplasmic compartments were estimated as 209.9, 84.9, and 95.3 μl g^–1^ FW. In *F. excelsior* with an average water content of 628 μl g^–1^ FW, the corresponding volumes were 450.3, 49.9, and 45.9 μl g^–1^ FW (Supplementary Table S2).

### Subcellular metabolite concentrations

Subcellular concentrations were calculated for each sugar based on the subcellular volumes and the subcellular sugar contents measured as described above. Sucrose was mainly concentrated in the cytoplasm (29.5–59.8mM), followed by the vacuole (9.5–35.9mM), and was lowest in the stroma (0.3–16.5mM; Table 2). The highest concentrations of glucose and fructose were found in the vacuole followed by the cytoplasm. In contrast, the highest concentration of mannitol was found in the stroma followed by the vacuole.

**Table 2.** Carbohydrate concentrations in the vacuolar, chloroplastic, and cytoplasmic compartments of leaves from Quercus robur and Fraxinus excelsiorThe evaluation is based on volumes of vacuolar, stromal, and cytoplasmic compartment shown in Supplementary Table S2 at *JXB* online.

	Concentration (mM)		
	Vacuole	Stroma	Cytoplasm
*Quercus robur*			
Mannitol	ND	ND	ND
Glucose and fructose	36.7±11.7	1.4±2.9	14.6±8.4
Sucrose	35.9±6.8	16.5±13.6	59.8±25.8
Raffinose	ND	ND	ND
Stachyose	ND	ND	ND
*Fraxinus excelsior*			
Mannitol	24.2±2.6	58.8±9.6	3.1±0.3
Glucose and fructose	6.7±6.1	6.6±11.5	17.4±22.6
Sucrose	9.5±5.0	0.3±0.5	29.5±20.6
Raffinose	0.8±0.3	1.7±0.7	0.0±0.0
Stachyose	2.2±0.8	5.3±1.9	0.0±0.0

ND, not detected.

### Phloem sap concentrations and concentration gradients

A comparison between the sugar concentrations in the cytosol of MCs and the phloem allows determination of the existence and direction of concentration gradients. The data that were obtained in this regard are summarized in Table 3. Phloem sap was collected by the laser–aphid–stylet technique. Using this method, it is possible to obtain pure phloem sap from intact plants. The two species differed greatly in their distribution of transport sugars. Sucrose was the major sugar in the phloem sap of *Q. robur* (Table 3). No or only traces of glucose and fructose were found in the phloem (data not shown). In *F. excelsior*, however, high amounts of oligosaccharides and mannitol were translocated in addition to sucrose (Table 3).

**Table 3.** Carbohydrate concentrations in the cytoplasm of mesophyll cells of leaves as well as in the apoplast and in the phloem sap from Quercus robur and Fraxinus excelsiorMean values of *n*=2–6 independent measurements are shown.

	Cytoplasm (mM)	Apoplast (mM)	Phloem sap (mM)	Phl/Apo ratio	Phl/Cyt ratio
*Quercus robur*					
Sucrose	59.8±25.8	0.4±0.2	1015±119	2538	17
Oligosaccharides	ND	ND	ND		
Mannitol	ND	ND	ND		
*Fraxinus excelsior*					
Sucrose	29.5±20.6	0.1±0.03	403±239	4030	14
Oligosaccharides	0.0±0.0	0.0±0.0	612±169	ND	ND
Mannitol	3.1±0.3	2.4±0.4	147±70	61	47

ND, not detected.

The sucrose concentration found in the phloem sap of *Q. robur* was very high (~1M; Table 3) whereas the sucrose concentration in the cytoplasm of MCs was much lower. Therefore, the gradient of sucrose between the phloem sap and the cytoplasm of MCs was 16-fold (Table 3). In *F. excelsior*, the sucrose concentration was also much lower in the cytoplasm of MCs than in the phloem sap, and the corresponding gradient of sucrose was 14-fold (Table 3).

### Osmotic pressure in leaf extracts and the phloem

**Fig. 2.**
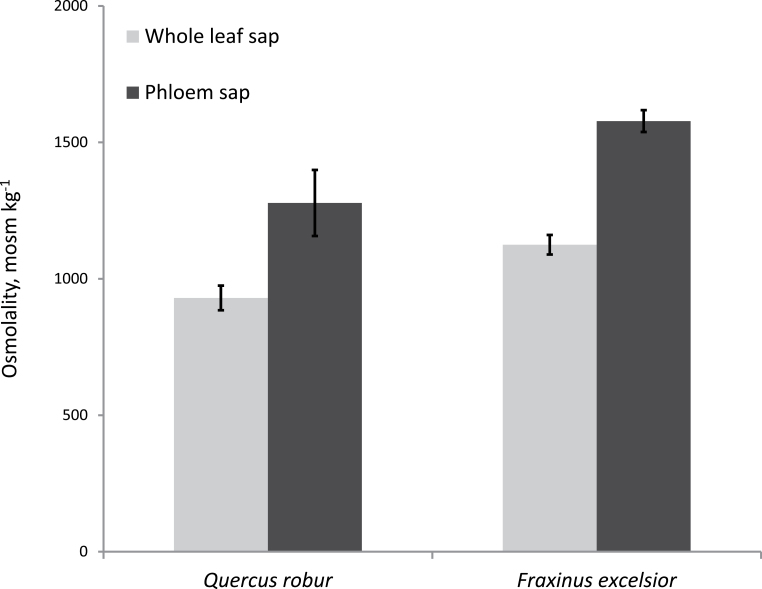
Osmolalities of whole-leaf sap and phloem sap of *Q. robur* and *F. excelsior*. Mean values from three independent measurements ±SD are shown. The data of phloem sap are based on the sugar concentrations in phloem sap shown in Table 3.

Plant species which are putative passive phloem loaders have higher whole-leaf osmolalities than active loading species (Fu *et al.*, 2011). Therefore, the osmolality of the whole-leaf sap and of the phloem sap was determined at the middle of the light period (Fig. 2). The values measured in whole-leaf saps were 930 mOsmol kg^–1^ for *Q. robur* and 1125 mOsmol kg^–1^ for *F. excelsior*. For determination of the osmotic pressure in the phloem sap, the approximate values were measured in solutes containing known concentrations of sugars in the phloem sap (summarized in Table 3). Because phloem sap also contained amino acids and different inorganic anions and cations, the real osmolality of phloem sap is probably higher. The values of phloem sap were 1278 mOsmol kg^–1^ for *Q. robur* and 1544 mOsmol kg^–1^ for *F. excelsior* (Fig. 2). These results show that in both species the osmolality of the phloem sap was always higher than that of whole-leaf sap.

The osmolalities of whole-leaf sap of different plant groups are shown in Supplementary Fig. S1 at *JXB* online. The osmolalities of trees were overall higher compared with those of herbs. They ranged from 340 mOsmol kg^–1^ to 590 mOsmol kg^–1^ in herbs and from 620 mOsmol kg^–1^ to 1150 mOsmol kg^–1^ in trees. The leaf water content of trees was between 54.4% and 67.5%, whereas in herbs it was between 74.2% and 88.7% (Supplementary Fig. S2). A negative correlation between leaf water content and osmolality was obvious (Supplementary Fig. S2).

### Isolation of sucrose transporters from source leaves of the two species

Type I SUTs have been shown to play an important role in apoplastic phloem loading in several species (Reinders *et al.*, 2012). As for potential apoplastic phloem loading pathways in *Q. robur* and *F. excelsior* that were indicated by the data obtained above, the putative SUTs involved in this process were isolated. Full-length sequences of SUTs were obtained by using degenerated primers followed by RACE-PCR from total RNA extracts from source leaves of *Q. robur* and *F. excelsior*. The sequences of *QrSUT1* (1.668bp, accession no. KF736982) and *FeSUT1* (1.869bp, accession no. KF736981) contained ORFs of 500 and 517 amino acids, corresponding to a calculated mol. wt of 53.2kDa and 54.2kDa, respectively. Topology predictions indicated that both proteins possess 12 membrane-spanning regions with a longer central loop.

**Fig. 3.**
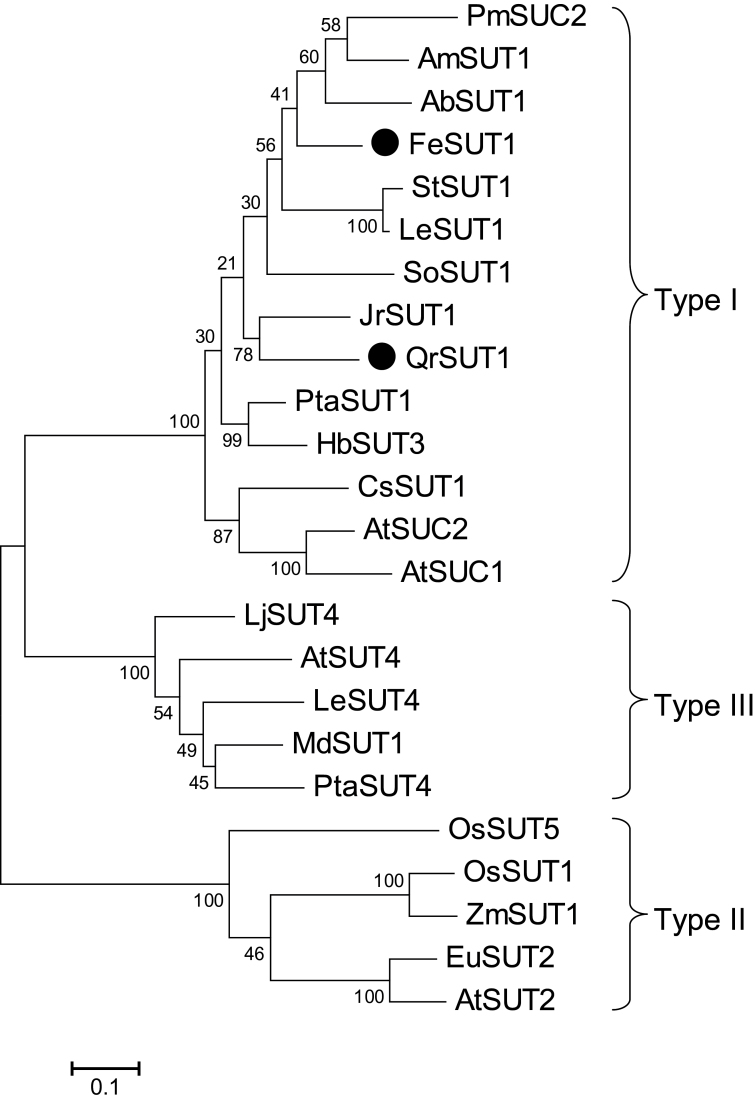
Phylogenetic analysis of sucrose transporters of plants with *QrSUT1* and *FeSUT1*. Protein alignment was performed using Clustal W within the MEGA5 software. The non-homologous variable C- and N-termini of the protein sequences were trimmed. A maximum likelihood tree with 100 bootstrap repetitions was generated using MEGA5 software. Numbers indicate percentage bootstrap analysis. The bar indicates evolutionary distance. *FeSUT1* (KF736981) and *QrSUT1* (KF736982) are marked with filled circles. *AmSUT1* (*Alonsoa meridionalis*; AF483211), *AbSUT1* (*Asarina barclaiana*; AAF04294), *AtSUC1*, *AtSUC2, AtSUT2, AtSUT4* (*Arabidopsis thaliana*; CAA53147, CAA53150, CAB92307, AAL59915), *CsSUT1* (*Citrus sinensis*; AAM29150), *DcSUT1a* (*Daucus carota*; CAA76367), *EuSUT2* (*Eucommia ulmoides*; AAX49396), *HbSUT3*, *HbSUT4* (*Hevea brasiliensis*; ABK60190, ABK60191), *JrSUT1* (*Juglans regia*; AAU11810), *LeSUT1*, *LeSUT4* [*Lycopersicon esculentum* (now renamed *Solanum lycopersicum*); CAA57726, AAG09270], *LjSUT4* (*Lotus japonicus*; CAD61275), *MdSUT1* (*Malus domestica*; AAR17700), *OsSUT1*, *OsSUT5* (*Oryza sativa*; BAA24071, BAC67165), *PmSUC2* (*Plantago major*; CAA53390), *PtaSUT1*, *PtaSUT4* (*Populus trichocarpa*; 18221401, HM749900), *SoSUT1* (*Spinacia oleracea*; CAA47604), *StSUT1* (*Solanum tuberosum*; CAA48915), *ZmSUT1* (*Zea mays*; BAA83501).

As shown in Fig. 3, phylogenetic analysis revealed high similarities of the amino acid sequences of the products of *QrSUT1* and *FeSUT1* to several other SUTs from herbaceous and tree species. SUT sequences form groups depending on their putative function and similarities on a family level, and therefore they are categorized into groups from type I to type III (Aoki *et al.*, 2003; Reinders *et al.*, 2012). Both transporters which were found in this study group into the type I subcategory. Type 1 SUTs play an essential role in apoplastic phloem loading and it has been proven that several SUT1 transporters are located at the plasma membrane of CCs (*PmSUC2*, Stadler *et al.*, 1995; *AtSUC2*, Stadler *et al.*, 1996; *AmSUT1*, Knop *et al.*, 2004; *LeSUT1*, *StSUT1*, Schmitt *et al.*, 2008). *FeSUT1* showed high sequence similarities to *AmSUT1* and *AbSUT1* from *Alonsoa meridionalis* and *Asarina barclaiana*, respectively. These species were reported to be putative mixed apoplastic and symplastic phloem loaders (Voitsekhovskaja *et al.*, 2006). *QrSUT1* formed a group with *JrSUT1* from *Juglans regia* (Juglandaceae), which is a member of the order Fagales, as is *Q. robur*.

## Discussion

### The subcellular distributions of sugars are similar in leaves of herbaceous and tree species

The non-aqueous fractionation technique enables the determination of metabolites in the vacuolar, stromal, and cytoplasmic compartment in the steady state of photosynthesis. For this analysis, a leaf was treated as a uniform metabolic compartment representing an average MC, subdivided into the three compartments listed above. This is obviously a simplification of a more complex pattern, but to date it is one of the best ways to obtain information about subcellular metabolite levels *in vivo*. This method provides highly reproducible results for metabolites that were exclusively confined to a single compartment. A higher variation was found for metabolites located in more than one compartment, and the variability was greatest when the proportion found in a particular compartment was low. In those cases, a formal evaluation of the standard deviations (SDs) illustrated the reproducibility of the data. Farré *et al.* (2001) estimated that the limit of detection of a compound in a particular compartment is ~5% of the total amount in the tissue. Non-aqueous fractionation with leaves of woody plant species was more difficult to perform than with herbaceous species. This can be seen, for example, for glucose and fructose. Hexoses are often confined to the vacuole (Riens *et al.*, 1991; Nadwodnik and Lohaus, 2008). In the case of *Q. robur* and *F. excelsior* this was true for part of the non-aqueous gradients, but in other gradients a portion of glucose and fructose was also found in the cytoplasm, which led to high standard deviations for the proportion of glucose and fructose in this compartment (Table 1). This finding was probably an artefact of the technique. However, Voitsekhovskaja *et al.* (2006) have shown that the concentrations of hexoses and sucrose in MCs measured either by a single-cell technique or by non-aqueous fractionation were similar.

While the method yields reasonable results for the subcellular distribution of mono- and disaccharides, it is less suitable for the determination of subcellular RFO concentrations. As RFOs were synthesized in ICs and the concentration in the phloem was at least one order of magnitude higher than in the MCs, it was to be expected that a considerable amount of RFOs associated with the MCs arose from the high content in the phloem. In this case, the method tends to overestimate somewhat concentrations in the mesophyll compartments. However, even overestimated subcellular concentrations would not affect the conclusions about the direction of diffusion gradients between the mesophyll and phloem as discussed below.

A comparison of the subcellular distributions of sugars in leaves of *Q. robur* and *F. excelsior* with those of other herbaceous plants showed several similarities. Hexoses were often confined to the vacuole in leaf cells of herbs (Riens *et al.*, 1991; Winter *et al.*, 1992; Voitsekhovskaja *et al.*, 2006; Nadwodnik and Lohaus, 2008). As shown in Table 1, these findings are also true for leaves of both tree species. The percentage of glucose and fructose found in the vacuole was between 83% and 85% (Table 1). On the other hand, between 53% and 73% of the sucrose detected in both tree species was located in the vacuole (Table 1). However, due to the smaller volume of the cytoplasm (Supplementary Table S2 at *JXB* online) relative to that of the vacuolar compartment, cytoplasmic sucrose concentrations were similar to or higher than those in the vacuole. A similar distribution has been reported for leaves of several herbaceous species (Riens *et al.*, 1991; Winter *et al.*, 1992; Voitsekhovskaja *et al.*, 2006; Nadwodnik and Lohaus, 2008). The main portion of mannitol in *F. excelsior* occurred in the vacuolar compartment, whereas the highest concentration was found in the stroma (Tables 1, 2). Similar results were shown for herbaceous plants such as *Anthirrinum majus*, *Petroselinum hortense*, *Asarina barclaiana*, or *Apium graveolens* (Moore *et al.*, 1997; Voitsekhovskaja *et al.*, 2006; Nadvodnik and Lohaus, 2008).

### Phloem loading mechanism in *Q. robur*

The pathway and mechanism of phloem loading in tree species that belong to the open type 1 (CC) minor vein subcategory (characterized by numerous plasmodesmata between the CC and adjacent cells) are still a matter of debate (Turgeon and Medville, 1998, 2004; Goggin *et al.*, 2001; Fu *et al.*, 2011; Slewinski *et al.*, 2013). Gamalei (1989) has assigned the members of the genus *Quercus* to the open minor vein type, and *Q. robur* was analysed as a representative of this group to resolve the question of whether the mechanism of phloem loading is active or passive. In view of the minor vein structure, members of the genus *Quercus* have one essential condition for passive transfer of sucrose into the phloem by diffusion and that is the abundance of symmetrically branched plasmodesmata at the CC–MC interface (Fig. 1C). *Quercus coccinea*, another species of this genus, was assigned to the group of passive phloem loaders by Fu *et al.* (2011) because this species translocated sucrose and has shown high leaf osmolality. Indeed, the leaf osmolality of *Q. robur* and other trees was always found to be higher than in herbs (Supplementary Fig. S1 at *JXB* online). The higher osmolality was correlated with the lower leaf water content of trees (Supplementary Fig. S2). The sugar content in the leaves of *Q. robur* (Table 1) was higher than or in some cases similar to the content in herbaceous species that were also measured in the second half of the light period (Riens *et al*., 1991, 1994; Knop *et al.*, 2001; Nadwodnik and Lohaus, 2008). However, the higher sugar contents in tree leaves cannot be the only reason for the higher osmolality in comparison with those of herbs. Additional solutes in tree leaves have to be taken into account to explain the increase of the overall osmolality. Fu *et al.* (2011) have shown that leaves of trees have higher concentrations of sugars as well as other polar metabolites than those of herbaceous plants. They have further assumed that the majority of trees (active and passive phloem loaders) require higher concentrations of foliar metabolites than herbs to maintain leaf turgor as a consequence of lower hydraulic conductance in trees.

Despite the high osmolality of the leaf sap of *Q. robur*, the osmolality of the phloem sap was much higher compared with the osmolality of the whole-leaf sap (Fig. 2). Furthermore, the concentration gradient of sucrose between the cytoplasm of MCs and the phloem was ~16-fold (Table 3). This value corresponds well to the gradient in other apoplastic phloem loaders (Lohaus *et al.*, 1995). Moreover, in tulip tree, the osmotic potential of minor vein phloem, estimated by plasmolysis, was much higher than that of the mesophyll, demonstrating the presence of a strong solute gradient (Goggin *et al.*, 2001) although the tulip tree belongs to the open type 1 (CC) subcategory similarly to *Q. robur*. In view of the higher osmolality in the phloem than in the MCs and the uphill concentration gradient of sucrose, it may seem doubtful that the transfer of sucrose from the MCs to the CCs is the result of passive diffusion. Instead active loading mechanisms are more probable.

Turgeon and Medville (2004) have shown that some tree species that belong to the open type I (CC) subgroup such as *C. barbinervis* and *L. styraciflua* were active phloem loaders and these tree species load the phloem via the apoplast, despite the fact that they had high plasmodesmatal counts in the minor veins. Furthermore, they showed that the abundance of plasmodesmata in the minor veins correlates positively with high plasmodesmal connections between MCs, and have concluded that this feature of leaf anatomy may not be directly related to phloem loading. The high plasmodesmatal frequency at the MC–CC interface in *Q. robur* and other tree species did not prevent active phloem loading (Goggin *et al.*, 2001; Turgeon and Medville, 2004). Evidence from these studies suggested active apoplastic phloem loading against a concentration gradient. Although it may seem contrary to expectations for a plant that loads via the apoplast to have many plasmodesmata between CCs and MCs, it is possible that the size exclusion limit at this interface restricts diffusion of sucrose or that the pores close in response to differences in turgor pressure between the two joined cell types (Oparka and Prior, 1992).

By means of reverse transcription–PCR (RT–PCR), full-length sucrose transporter cDNAs were obtained from source leaves of *Q. robur* (*QrSUT1*; KF736982) and sequence comparison showed high homologies with known plant SUTs that are involved in phloem loading of sucrose (Fig. 3). Although final proof is still missing, it seems possible that a SUT is also involved in the phloem loading of *Q. robur*.

### Phloem loading mechanism in *F. excelsior*

The minor veins of *F*. *excelsior* contained ICs, which are typical for RFO-translocating species (Fig. 1D). In addition, the minor veins also contained one or two ordinary CCs (Fig. 1D). This diversity of CCs in the minor veins is also typical for RFO-translocating species and the putative symplastic phloem loaders analysed so far (Fisher, 1986; van Bel *et al.*, 1992; Turgeon *et al.*, 1993; Knop *et al.*, 2001).

To date there have been few data available dealing with concentrations of sucrose, sugar alcohols, and RFOs in the phloem sap of trees. The total concentration of sugars in the phloem sap of *F. excelsior* was ~1.2M, which was similar to the sucrose concentration in apoplastic loaders (~0.8–1.5M; Riens *et al.*, 1991; Lohaus *et al.* 1995; Lohaus and Möllers, 2000) and to the total sugar concentration in symplastic or mixed phloem loaders (~0.8–1.3M; Knop *et al.*, 2001).

RFOs accounted for ~70% of the carbon exported from the leaf in *F. excelsior*, sucrose for 25%, and mannitol for ~5% (Table 3). In this case, sugar alcohols were not the main export form for carbon as in other tree species, which translocate only sucrose and sugar alcohols. In peach (*Prunus persica*), sorbitol accounted for ~70% of the exported carbon (Nadwodnik and Lohaus, 2008). In *F. excelsior*, the concentration gradient for mannitol between the cytoplasm of MCs and the phloem was ~47-fold (Table 3). Nadwodnik and Lohaus (2008) have shown lower or similar concentration gradients for sugar alcohols (2- to 40-fold) in *Plantago major*, *Plantago maritima*, *Apium graveolens*, and *P. persica* which probably exhibit an active phloem mode. The lowest gradient was found in *P. persica* because the cytoplasmic concentration of sugar alcohols was very high (Nadwodnik and Lohaus, 2008). For several species, polyol transporters were cloned (*P. major*, Ramsperger-Gleixner *et al.*, 2004; *Malus domestica*, Watari *et al.*, 2004) and the function of the putative transporter proteins in phloem loading was corroborated by their specific localization in the minor phloem (Ramsperger-Gleixner *et al.*, 2004; Watari *et al.*, 2004). Unfortunately, no data of transporters exist for *F. excelsior*. In contrast, when leaf discs of *Asarina* ssp. or *M. domestica* were provided with exogenous [^14^C]mannitol or [^14^C]sorbitol, the radiolabel does not accumulate in the minor veins as would be expected if they load actively by transporters (Reidel *et al.*, 2009). From these results, it was concluded that in these species sorbitol was loaded symplastically and passively (Reidel *et al.*, 2009). However, this loading mode seems unlikely for *F. excelsior* because the steep uphill concentration gradient for mannitol strongly suggests active transport systems. In *F. excelsior*, mannitol has access to the ICs from the MCs through plasmodesmata because mannitol is much smaller than sucrose. However, this is not an explanation for the uphill gradient because no ‘polymer trapping’ exists for sugar alcohols as it does for RFOs (Turgeon and Gowan, 1990). *Fraxinus excelsior* contains two different kinds of CCs (Fig. 1). Perhaps mannitol might be loaded actively into the ordinary CCs whereas the function of the ICs is mainly in the synthesis of RFOs. The possible existence of multiple loading modes in the same vein raises interesting questions concerning the coordination of transport rates of the different compounds (Reidel *et al.*, 2009).

*Fraxinus excelsior* has a structural potential for symplastic phloem loading of assimilates, as indicated by the presence of plasmodesmal fields in its ICs. Furthermore, it translocated high amounts of RFOs (Table 3), which is also a characteristic of putative symplastic phloem loaders. Earlier studies with Curcubitaceae and *A. meridionalis* indicated that stachyose is probably synthesized in the ICs (Holthaus and Schmitz, 1991; Beebe and Turgeon, 1992; Voitsekhovskaja *et al.*, 2009), which probably also applies to *F. excelsior*.

In agreement with the polymer trap model, sucrose is expected to be present in the cytosol of MCs of symplastic loading species in higher concentrations than in the phloem. However, in *F. excelsior*, the sucrose concentration in the phloem sap was ~14-fold higher as in the cytoplasm of MCs (Table 3). This value corresponds well to the values found in apoplastic phloem loaders that were ~5- to 30-fold (Lohaus *et al.*, 1995; Lohaus and Möllers, 2000; Knop *et al.*, 2001), but it is much higher than the 2-fold concentration gradient for sucrose in the RFO-translocating species *A. meridionalis* (Scrophulariaceae) (Voitsekhovskaja *et al.*, 2006). In view of the concentration gradient of sucrose, it may seem unlikely that the transfer of sucrose from the MCs to the phloem is the result of a passive diffusion. Rather, phloem loading in *F. excelsior* is an active process. SUT cDNAs were also obtained from source leaves of *F. excelsior* (*FeSUT1*, KF736981). Although final proof is still missing, it seems possible that a SUT is also involved in the phloem loading in this RFO-translocating species. Therefore, it is reasonable to assume that at least some of the sucrose loads via the apoplastic pathway. Furthermore, the data show that sucrose would diffuse back into MCs rather than enter the phloem, if the opening of plasmodesmata in ICs was not regulated in *F. excelsior.* The degree of the turgor pressure difference might be the mechanism regulating opening/closure of the plasmodesmata (Oparka and Prior, 1992).

The mixed phloem loader *A. meridionalis* contained two types of CCs in the minor veins, ordinary CCs as well as ICs (Knop *et al.*, 2001). The SUT protein was only present in the ordinary CCs whereas the stachyose synthase gene was expressed only in ICs (Voitsekhovskaja *et al.*, 2009). It could be assumed that the different function of the CCs is similar to the situation in *F. excelsior* because these two types of CCs were also present (Fig. 1). It seems likely that *F. excelsior* employs multiple loading strategies, even in the same vein.

Slewinski *et al.* (2013) have discussed that perhaps all plant species use more than one phloem loading mechanism, even if one mechanism is dominant. It was observed that in cucurbits, which are categorized as symplastic loaders, a shift in the sucrose to stachyose ratio occurred after an infection with *Cucumber mosaic virus* (CMV) and an apoplastic loading pathway was activated (Gil *et al.*, 2011). This indicates that phloem loading is highly affected by environmental influences. The existence of heterogeneous phloem loading mechanisms in one plant species may provide a more flexible physiological reaction of the plant to changing environmental conditions. Perhaps this is also the case for *F. excelsior*.

## Conclusion

Data presented here suggest that at least portions of phloem loading happen to be active in both *F. excelsior* and *Q. robur*. The measured sugar contents in leaves and leaf exudates were in accordance with previous studies (Zimmermann and Ziegler, 1975); however, it was the first time that actual sugar concentrations in cell compartments and phloem sap were compared. An uphill concentration gradient for sucrose between the cytoplasm of MCs and the phloem was shown for both tree species. *Quercus robur* tends to be an apoplastic phloem loader, whereas *F. excelsior* seems to be a mixed apoplastic–symplastic phloem loader. Further studies need to be carried out in order to clarify the exact loading mechanisms. Since putative SUTs were obtained, immunolocalization experiments could give an insight into the role of these transporters in phloem loading.

## Supplementary data

Supplementary data are available at *JXB* online.

Figure S1. Osmolality of leaves of different plant groups.

Figure S2. The leaf water content and the osmolality of the leaf sap of the different plant groups.

Table S1. Relative volumes (%) of the subcellular compartments to the total volume of mesophyll cells from *Quercus robur* and *Fraxinus excelsior*.

Table S2. Dry weight (*n*=6), water and gas space (*n*=6), and volumes of subcellular compartments of mesophyll cells of leaves from *Quercus robur* and *Fraxinus excelsior*.

Supplementary Data

## Abbreviations:

BSC: bundle sheath cell
CC: companion cell
IC: intermediary cell
MC: mesophyll cell
RFO: raffinose oligosaccharide
RT–PCR: reverse transcription–PCR
SUT: sucrose transporter
TC: transfer cell.
