# Supplementary material for: Apoplastic and symplastic phloem loading in Quercus robur and Fraxinus excelsior
Source: J Exp Bot. 2014 Mar 3;65(7):1905–16. doi: 10.1093/jxb/eru066 (PMC3978624; doi:10.1093/jxb/eru066)
Supplement: Supplementary Data [file supp_65_7_1905__index.html]

Apoplastic and symplastic phloem loading in Quercus robur and Fraxinus excelsior — Apoplastic and symplastic phloem loading in Quercus robur and Fraxinus excelsior — Supplementary Data 

# Apoplastic and symplastic phloem loading in *Quercus robur* and *Fraxinus excelsior*

## Supplementary Data

Data files

**Files in this Data Supplement:**

- Supplementary Data - Supplementary Data
