# Supplementary material for: Apoplastic and symplastic phloem loading in Quercus robur and Fraxinus excelsior
Source: J Exp Bot. 2014 Mar 3;65(7):1905–16. doi: 10.1093/jxb/eru066 (PMC3978624; doi:10.1093/jxb/eru066)
Supplement: Supplementary Data [file supp_eru066_jexbot113589_file001.pdf]

Journal of Experimantal Botany

Apoplastic and symplastic phloem loading in *Quercus robur* and *Fraxinus excelsior*

Soner Öner-Sieben, Gertrud Lohaus

Supplementary Data

Table S1. Relative volumes (%) of the subcellular compartments at the total volume of mesophyll cells from *Quercus robur* and *Fraxinus excelsior*. Data were obtained from morphometric analysis (n = 30-38). The total volume of a mesophyll cell is defined as 100 %. Cytoplasm is defined as cytosol, peroxisomes, mitochondria and endoplasmatic reticulum. Mean values ± SD are shown.

	Q. robur	*F. excelsior*
Vacuole (%)	39.3 ± 14.0	71.7 ± 6.4
Chloroplast (%)	31.8 ± 10.0	15.9 ± 5.4
Nucleus (%)	7.8 ± 3.1	3.9 ± 1.1
Cytoplasm (%)	21.0 ± 6.6	8.6 ± 3.3

Table S2. Dry weight (n = 6), water - and gas space (n = 6), and volumes of subcellular compartments of mesophyll cells of leaves from *Quercus robur* and *Fraxinus excelsior*. Subcellular volumes were calculated from relative volumes of the subcellular compartments (Table S1) and the water space. The water space was defined as fresh weight minus dry weight. The gas space of the leaves was defined as space outside of the symplast and the liquid apoplast and corresponds to the gaseous intercellular space.

	Q. robur	*F. excelsior*
Dry weight (mg g^{-1} FW)	466 ± 85	372 ± 120
Water space (µl g^{-1} FW)	534 ± 90	628 ± 161
Gas space (µl g^{-1} FW)	433 ± 42	521 ± 47
Vacuole (µl g^{-1} FW)	209.9	450.3
Stroma (µl g^{-1} FW)	84.9	49.9
Cytoplasm (µl g^{-1} FW)	95.3	45.9

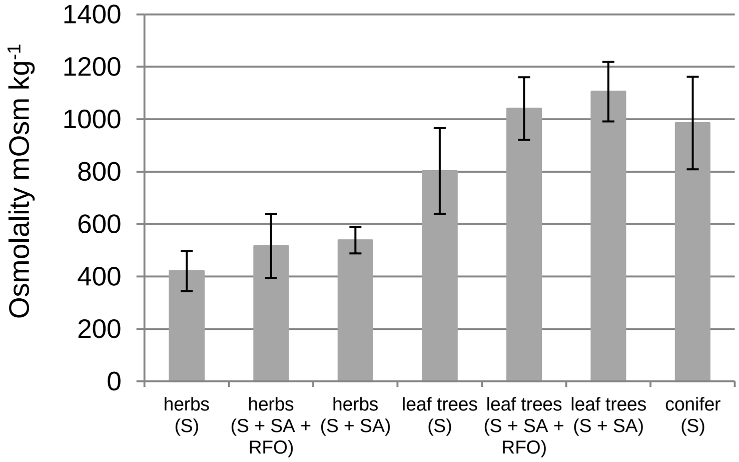

Fig. S1. Osmolality of leaves of different plant groups. The leaves contain different amounts of sucrose, raffinose-oligosaccharides and/or sugar alcohols. herbs (S): leaves of herbaceous plant containing sucrose (*Beta vulgaris, Brassica napus, Helianthus annuus, Pisum sativum, Vicia faba, Zea mays, Hordeum vulgare*); herbs (S + SA + RFO): leaves of herbaceous plant containing sucrose, sugar alcohols and raffinose-oligosaccharides (*Ajuga reptans, Alonsoa meridionalis, Asarina barclaiana, Lophospermum erubescens, Cucurbita maxima, Lamium album, Verbascum*); herbs (S + SA): leaves of herbaceous plant containing sucrose and sugar alcohols (*Plantago major, Apium graveolens*); leaf trees (S): leaves of tree species containing mainly sucrose (*Fagus sylvatica, Quercus robur, Acer campestre*); leaf trees (S + SA + RFO): leaves of tree species containing sucrose, sugar alcohols and raffinose-oligosaccharides (*Fraxinus excelsior, Syringa vulgaris*); leaf trees (S +SA): leaves of tree species containing sucrose and sugar alcohols (*Malus domestica, Prunus avium, Prunus persica*); conifers (S): leaves of conifers containing sucrose (*Abies grandis, Picea abies, Pinus sylvestris*).

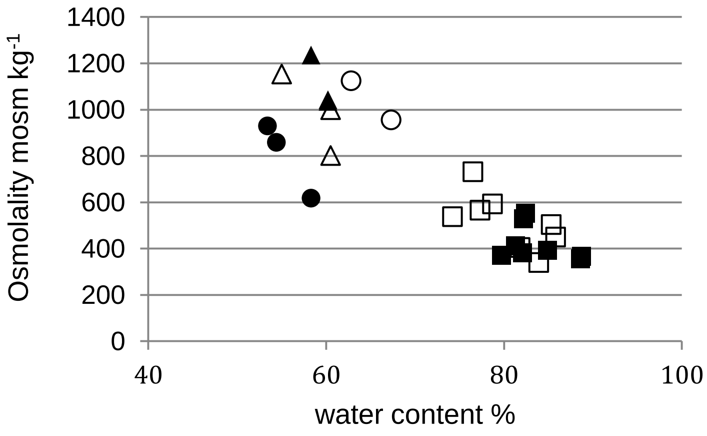

Fig. S2. The leaf water content and the osmolality of the leaf sap of the different plant groups (the same plant species shown in Fig. S1). Closed squares: herbs (S) and (S + SA), open squares: herbs (S + SA + RFO), closed circles: leaf trees (S), open circles: leaf trees (S + SA + RFO), closed triangles: leaf trees (S + SA), open triangles: conifers.
